# From work to community: how social participation and retirement can benefit older adults’ depression

**DOI:** 10.3389/fpsyt.2025.1522222

**Published:** 2025-03-03

**Authors:** Yiyu Zhan, He Ning, Yuchen Zhang

**Affiliations:** Department of Sociology, Sungkyunkwan University, Seoul, Republic of Korea

**Keywords:** retirement, social participation, CHARLS, depression, mediating analysis

## Abstract

**Objectives:**

The retirement phase and social participation influence the depressive symptoms of older individuals. Nonetheless, the fundamental mechanisms connecting these factors remain unclear. This analysis examined mediating social participation in the relationship between older adults’ retirement and depressive symptoms.

**Methods:**

The study analyzed 9,103 Chinese individuals aged 60 and above utilizing the 2020 China Health and Retirement Longitudinal Study (CHARLS). Researchers evaluated participants’ depression utilizing the Center for Studies in Epidemiology Depression Scale (CESD-10). The hierarchical multiple regression models were utilized to evaluate the link between retirement and depression, through cross-sectional analyses, along with the possible influence of social participation on this relationship.

**Results:**

Retirement significantly reduce older individuals’ depression and improves their mental health state. In addition, social participation as a mediating variable further enhanced the beneficial impacts of retirement on the mental health state.

**Conclusion:**

This research discovered that retirement indirectly influences older adults’ depression, with social participation playing a significant impact. Thus, it establishes a foundation for mitigating psychological issues in this demographic. Engaging in or sustaining social activities during later life enhances the mental well-being of older individuals. Promoting social participation among older individuals is a viable approach to reduce depression and facilitating successful aging initiatives in this demographic.

## 1 Introduction

In recent decades, China’s elderly population has shown a rapid and advanced aging trend (1) and is among the fastest-aging nations globally (2). By 2050, the percentage of the population individuals reaching 60 years and above in China is projected to climb from 11.47% in 2019 to 31.79%, while the old-age dependency ratio is forecast to rise from 0.17 to 0.52 (3). As people’s healthy life expectancy continues to grow (3), the number of retirees continues to rise, resulting in substantial financial burdens on both government and business budgets and creating labor shortages. Many nations are discussing the potential increase in retirement age due to an aging population, which is putting pressure on the resources of state pension schemes (4). However, the success of policies to delay the retirement age will largely depend on their influence on the healthful behaviors of older individuals.

As China enters an aging society, rapid urbanization, changes in family structure, and the impact of the one-child policy have led to a gradual weakening of the traditional family support network for older adults, separation of the elderly from their children (5), and a rapid increase in the number of empty nesters and elderly living alone. At the same time, rapid socioeconomic changes in China have also had a profound impact on the mental health of older adults (5), making them more vulnerable to mental health problems such as loneliness and depression (6). Depression is common among the elderly population. The mental health of the Chinese population continues to be challenged throughout the life course. As older adults experience a deterioration in physical functioning with age, they also face situations such as retirement and widowhood, which contributes to a continued increase in depressive symptoms with age (7, 8). A classic example is “geriatric depression” (9–11). In recent years, older adults’ depression has emerged as a significant social challenge, garnering heightened attention from the government, academics, mental health care groups, and others.

Retirement is an important turning point in an individual’s life and therefore has a potential impact on depression (12) as it may disrupt social support networks but also terminate stressful working conditions (13). As a result, most scholars, both nationally and internationally, have indicated that the influence of retirement on depression is disputable. For example, some studies have found negative effects. Retirement signifies a challenging adjustment, such as that resulting from the loss of job functions, reduced contact with coworkers, and a marked decrease in income (14), which disrupts an individual’s established routine of daily life (15) and thus negatively affects health (16, 17). While other studies have found positive effects of retirement on depression (18). Role theory suggests that it entails disengaging from professional responsibilities, alleviating work-related stress or adverse working environments (19), and enabling older adults to pursue their interests, leisure activities, or physical exercise, thereby improving health (20, 21). For example, Mandal and Roe (2008), using the first six waves of the HRS, found that retirement reduced levels of depressive symptoms. Kolodziej and García-Gómez (2017) reported similar results (22). Using a discontinuous regression approach, Eibich discovered that retirement could improve an individual’s depression by alleviating associated work stress and anxiety, extending sleep duration, and promoting increased leisure activities (23). Moreover, retirement improves self-assessed health and reduces the risk of depression (24). And, voluntary retirement is linked to enhanced short-term health (25). In addition, retirement affects people’s schedules and everyday existence (26), offering increased possibilities for physical activity (27). Retirement is a period of reinvention of the self and enhancement of physical and mental health as they are free from work pressure and can have greater freedom in their time and activities (28), which allows for more social participation. Furthermore, when the health condition of people over 60 evolves, their roles in society also transform. They must achieve social participation in retirement while adapting to these significant transformations and maintaining average health amidst the steady reduction of resources (29).

Social participation is characterized as participation in social events (30), including paid and non-remunerated work, volunteering, and involvement in social groups that facilitate connection within the community (31). Social participation serves as a preventive factor that decreases adverse mental health disorders in older adults, including the possibility of functional limitations (30), cognitive decline (32), and depression (33). As individuals’ occupations are deeply linked to their social interactions and time management (34), social participation significantly connects with retirement. Participation in social activities post-retirement may increase retirement’s positive impacts on depression (35). For example, regular participation in social activities helps decrease symptoms of depression (36–38). Meanwhile, continuity theory suggests that as society evolves, traditional employment roles become less important to older people, while retirement is increasingly recognized as a meaningful and desirable life stage, providing opportunities for continued engagement in new roles such as social participation and leisure (39). Also, activity theory argues that people over 60 may increase their participation in society to counter the decline of social roles resulting from their retirement from work (40). Moreover, retirees participated more in volunteering, social events, and education or training programs (41) and sustained social participation is strongly associated with a reduction in depressive symptoms (42). This positive social participation can lead to accomplishment, self-worth, and social satisfaction.

### 1.1 Chinese policy

Since the 1950s, China has had a compulsory retirement system, whereby most workers, except for special types of work, must go through retirement procedures when they reach the legal retirement age. This policy has reduced the differences in retirement age due to personal choice or health status, resulting in a more concentrated retirement time, thus providing ideal conditions for studying the cause-and-effect relationship between retirement and depression. In contrast to most developed countries, China imposes fewer limitations on retirees’ ability to remain employed post-retirement. Retirees may re-enter the workforce in many capacities while concurrently earning their pensions. This system has so far remained substantially unchanged, despite the dramatic socio-economic and structural changes that have taken place over the past 30 years.

Overall, research indicates that retirement behavior greatly impacts the depression of older individuals; nonetheless, scholarly studies on the issue indicate two fundamentally different viewpoints. Early research indicated that retiring negatively impacted individual mental well-being. However, more recent studies indicate that retirement facilitates improvements in depression. Thus, there is less agreement regarding the effects of retiring on individual depression, whether positive or negative. *Secondly, the limited research to date has focused on systems where retirement age is flexible, whereas this study benefits from a relatively fixed retirement system.* Because China has a compulsory retirement system, and varying retirement regimes may result in disparate health outcomes associated with retirement (43). Secondly, the limited research to date has focused on systems where retirement age is flexible, whereas this study benefits from a relatively fixed retirement system. Because China has a compulsory retirement system, and varying retirement regimes may result in disparate health outcomes associated with retirement (43). Third, prior research has concentrated on how participation in society may reduce the negative effects of retirement. In contrast, there are fewer studies on whether post-retirement social participation is a pathway for older adults’ retirement to influence their mental well-being. Therefore, this analysis utilized 2020 CHARLS data, integration of social change in China to examine the relationship between work status differences and depression variations among older individuals in China and whether this relationship can be mediated through social participation. We therefore proposed the hypothesis:

H1: Retirement has a strong beneficial impact on older adults’ depression in China.

H2: Social participation significantly mediates the association between retirement and depression in Chinese older adults.

## 2 Research methods

### 2.1 Data sources

We used the latest cross-sectional data from the 2020 CHARLS (44). The China Health and Retirement Longitudinal Study (CHARLS) is the inaugural nationally representative study of older individuals, modeled after the U.S. Health and Retirement Study (HRS), to examine the health of China’s elderly population (45). The CHARLS national baseline survey was conducted in 2011-2012, with Wave 2 in 2013, Wave 3 in 2015, Wave 4 in 2018, and Wave 5 in 2020. The CHARLS sample was representative of those aged 45 years and older. A stratified multistage PPS random sampling strategy was used (46). This is a long-term survey conducted by the National Institute of Development Studies at Peking University, all with ethical approval from the Peking University Institutional Review Board. Since this study involved secondary analysis of anonymized data, no additional ethical approval was required. The questionnaire addresses fundamental personal data, family composition and economic help, health condition, physical metrics, healthcare usage and insurance, job status, retirement and pensions, finances, spending, assets, and community-related data. Therefore, this paper cleaned the data extracted from CHARLS 2020 to exclude responses with outliers or missing values in selected key variables after selecting the necessary variables from the database that fit the study design to obtain 9,103 adult respondents over 60 for further modeling.

### 2.2 Measures

#### 2.2.1 Depression

The CES-D scale is specifically designed to assess depressive symptoms (47), but a clinical diagnosis of major depressive disorder (MDD) was not made in this paper. The CES-D scale in the CHARLS2020 survey assesses respondents’ psychological condition over the last week across ten factors, eight of which are considered harmful (e.g., I feel depressed), and two of them are positive (e.g., I am hopeful about the future). “Little or no time,” “some or little time,” “occasional or moderate time,” and “most or all the time” were the four choices for each. The four choices have been assigned whole numbers ranging from 0 to 3, with the values for the affirmative questions inverted. The overall mental health score varied from 0 to 30, with higher levels correlating with increased depression (48).

#### 2.2.2 Retirement

According to definitions provided by prior literature (49, 50), we describe a person who retires as someone who has either retired or resigned from employment (from an organization, institution, or business) as defined by the questionnaire “Have you ever retired from work?” to obtain. Following data analysis, retirement is identified as ‘1’ and non-retirement as ‘0’.

#### 2.2.3 Social participation

According to a prior study (51, 52), the CHARLS2020 questionnaire evaluated social participation by asking, “In the past month, have you done any of the following activities?” listing seven types of social activities: (a) interacted with friends, (b) played mah-jongg, played chess, played cards, or went to community club, (c) provided help to family, friends, or neighbors who do not live with you and who does not pay for your help, (d) went to a sports, social, or other kind of club, (e) took part in a community-related organization, (f) done voluntary or charity work, and (g) cared for a sick or disabled adult who does not live with you and who does not pay for your help. A score ranging from 0 to 7 is obtained by allocating one point to each action. A higher score signifies a greater degree of social participation.

#### 2.2.4 Sociodemographic variables

Furthermore, to reduce the impact of personalities of the research population and other environmental influences on the results and to enhance the consistency of the regression outcomes, in this paper, referring to Coe and Zamarro’s study, we control for several demographic and sociological characteristics variables. The age range of adults is 60 to 120 years. Next are sex (female = 0, male = 1), marital status (never married, divorced, widowed = 0, married (with a spouse), cohabiting = 1), educational attainment (below elementary school = 0, elementary school = 1, middle school = 2, high school and above = 3), and household type (urban = 0, rural = 1). In addition, three health-related covariates were controlled for smoking (yes = 1, no = 0), drinking (yes = 1, no = 0), cardiovascular disease (yes = 1, no = 0) as well as physical activity (yes = 1, no = 0). Since data on income after retirement is limited, we use whether a person receives a pension (yes = 1, no = 0) as a proxy indicator. See **Table 1** for variable definitions (49).

**Table 1:** Descriptive statistics for variables, China health and retirement longitudinal study [mean ± SD/*n* (%)].

Variables	All (n = 9103)	Not retirement (n = 6,801)	Retirement (n = 2,302)	P^a^
Mental health	9.14 ± 6.54	9.991 ± 6.60	6.645 ± 5.65	<0.001
Social participation	0.67 ± 0.92	0.584 ± 0.83	0.94 ± 1.12	<0.001
Age	70.05 ± 7.2	69.99 ± 7.18	70.2 ± 7.23	0.182
Gender				<0.001
Male	4,550 (49.98%)	3,179 (46.74%)	1,371 (59.56%)	
Female	4,553 (50.02%)	3,622 (53.26%)	931 (40.44%)	
Education				<0.001
Less than elementary school	4,367 (47.97%)	3,957 (58.18%)	410 (17.81%)	
Elementary school	1,954 (21.47%)	1,482 (21.79%)	472 (20.50%)	
Middle school	1,657 (18.2%)	976 (14.35%)	681 (29.58%)	
High school and above	1,125 (12.36%)	386 (5.68%)	739 (32.10%)	
Marital status				<0.001
Married	7,335 (80.58%)	5,393 (79.30%)	1,942 (84.36%)	
Others	1,768 (19.42%)	1,408 (20.705)	360 (15.64%)	
Household type				<0.001
Rural	6,574 (72.22%)	6,124 (90.05%)	450 (19.55%)	
Urban	2,529 (27.78%)	677 (9.95%)	1,852 (80.45%)	
Pension				<0.001
Yes	7,961 (87.45%)	5,851 (86.03%)	2,110 (91.66%)	
No	1,142 (12.55%)	950 (13.97%)	192 (8.34%)	
Exercise				<0.001
Yes	8,125 (89.26%)	5,982 (87.96%)	2,143 (93.09%)	
No	978 (10.74%)	819 (12.04%)	159 (6.91%)	
Drink				<0.001
Yes	3,155 (34.66%)	2,199 (32.33%)	956 (41.53%)	
No	5,948 (65.34%)	4,602 (67.67%)	1,346 (58.47%)	
Smoke				0.304
Yes	2,366 (25.99%)	1,761 (25.89%)	605 (26.28%)	
No	6,737 (74.01%)	5,040 (74.11%)	1,697 (73.72%)	
Cardiovascular disease				0.017
Yes	7,830 (86.02%)	5,823 (85.62%)	2,007 (87.19%)	
No	1,273 (13.98%)	978 (14.38%)	295 (12.81%)	

a used independent t-test and Chi-square test.

### 2.3 Statistical analysis

Data has been examined with Stata 18.0. First, demographic variables were statistically analyzed using descriptive statistics, and an independent samples *t-test* and chi-square test were used to compare differences between groups of different sociodemographic characteristics. Second, the factors affecting older people’s depression were investigated using hierarchical multiple regression modeling. In the hierarchical regression model, Model 1 examines the effect of demographic control variables on depression. Model 2 examines the relationship between retirement and depression. Model 3 focuses on the relationship between retirement and social participation. Model 4 uses retirement and social participation as predictors of depression to test the mediating effect of social participation. The significance standards for all statistical analyses were established at p < 0.05. The bootstrap test was used to confirm the mediation effect and construct the mediating effect framework. The importance of the indirect effect was validated using 95% bias-corrected confidence intervals derived from 5,000 Bootstrap samples (53). Indirect effects were regarded as valid if the confidence interval excluded “0”, and the significance level was established when p < 0.05 (54).

## 3 Results

### 3.1 Demographic characteristics of subjects

The study included 9,103 older individuals, 6,801 non-retired, and 2,302 retired. The depression levels of the retired were generally lower than those of the non-retired (p < 0.001), and the retired also participated more in society. 49.98% of the participants were male, ranging from 60 to 120 years of age, with an average age of 70.05 years. Within the group of respondents, 69.44% had elementary school education or less, 72.22% lived in rural households, 80.58% were married, and 87.45% of the older adults had pensions and old-age insurance. Meanwhile, most of the older adults suffered from cardiovascular disease (86.02%), but a few of them smoke (25.99%) drank alcohol (34.66%). 89.26% of them exercised regularly. The characteristics of retired older adults include a high social participation score, a low depression score, being male, urban, less educated, married, having pension insurance, exercising regularly, and having underlying diseases (P < 0.05) (**Table 1**).

### 3.2 Correlation between retirement, social participation, and depression

**Table 2** indicates that retirement had a positive correlation with social participation (rs = 0.117, P < 0.001) and a negative association with depression (rs = -0.223, P < 0.001). Also, social participation negatively affected depression (rs = -0.059, P < 0.001).

**Table 2:** Correlation analysis of retirement, social participation, and depression.

	Retirement	Social Participation	Depression
Retirement	1.000		
Social Participation	0.117***	1.000	
Depression	-0.223***	-0.059***	1.000

***P<0.001.

### 3.3 The mediating effect of social participation on retirement and depression

Retirement and depression are strongly correlated. However, it is unclear how social participation in retirement and older adults’ depression relate to one another (35). To clarify this transmission mechanism, a sequential recursive model was established to examine the mediating effect of social participation, the mediating effect analysis given by Baron and Kenny (55). **Table 3** presents the findings regarding the mediating influence of social participation. Model 1 contained just demographic control factors, indicating that all variables, except age, pension, and smoking behavior, significantly influenced depression. In Model 2, retirement reduced depression in older adults at the 0.1% significance level (β = -1.771, p < 0.001), implying that older individuals who were retired had less of an increase in depression. In Model 3, retirement behavior markedly affected the social participation of the elderly at the 0.1% significance level (β = 0.159, p < 0.001), showing that retirement behavior increased the social participation of older individuals. In Model 4, both retirement behavior and social participation significantly negatively impacted depression in older individuals at the 0.1% level of significance, with the coefficient for retirement behavior showing a reduction compared to that in Model 1 (β = -1.725 & β = -0.250, p < 0.001), showing that social participation serves as a mechanism with which retirement influences depression in older individuals, indicating that social participation mediates the relationship between retirement behavior and the older adults’ depression (**Table 3**). Among the covariates, being male (β = -1.478, p < 0.001), never married, divorced, widowed (β = -1.372, p < 0.001), education (β = -0.793, p < 0.001), physical activity (β = -0.828, p < 0.001), and drinking behavior (β = -0.653, p < 0.001) were associated with better mental well-being. However, cardiovascular disease (β = 2.548, p < 0.001) correlated with worse depression in older individuals.

**Table 3:** Hierarchical multiple regression analysis of mediating of social participation (N = 9,103).

Variables	Model 1	Model 2	Model 3	Model 4
Retirement (ref. no)		-1.771*** (-8.33)	0.159*** (5.73)	-1.725*** (-8.11)
Social participation (0 - 7)				-0.250*** (-3.56)
Age (60 – 120)	-0.007 (-0.70)	0.001 (0.08)	-0.009*** (-6.78)	0.001 (-0.10)
Gender (ref. female)	-1.478*** (-8.97)	-1.437*** (-8.75)	-0.157*** (-7.50)	-1.477*** (-8.98)
Education (ref. < elementary school)				
Elementary school	-0.793*** (-4.55)	-0.702*** (-4.04)	0.102*** (4.53)	-0.678*** (-3.90)
Middle school	-1.546*** (-8.09)	-1.269*** (-6.57)	0.192*** (7.46)	-1.222*** (-6.31)
High school and above	-2.445*** (-10.63)	-1.967*** (-8.32)	0.342*** (10.94)	-1.874*** (-7.88)
Marital status (ref. not married)	-1.372*** (-7.91)	-1.295*** (-7.49)	-0.049* (-2.27)	-1.307*** (-7.56)
Household type (ref. urban)	1.840*** (1.67)	0.816*** (4.09)	-0.088*** (-3.40)	0.794*** (3.98)
Pension (ref. no)	0.038 (0.19)	0.090 (0.46)	0.074** (3.06)	0.112 (0.57)
Exercise (ref. no)	-0.828*** (-3.92)	-0.793*** (-3.76)	0.337*** (13.79)	-0.715*** (-3.38)
Drink (ref. no)	-0.653*** (-4.37)	-0.645*** (-4.34)	0.160*** (8.27)	-0.604*** (-4.04)
Smoke (ref. no)	0.212 (1.25)	0.200 (1.18)	0.078*** (3.55)	0.217 (1.28)
Cardiovascular disease (ref. no)	2.548*** (13.57)	2.557*** (13.67)	0.058* (2.41)	2.573*** (13.77)
_cons	9.630*** (10.74)	9.927*** (11.10)	0.848*** (7.80)	10.132*** (11.32)
N		9103.000	9103.000	9103.000
r2_a		0.118	0.079	0.120

t statistics in parentheses. Ref., reference. *p < 0.05, **p < 0.01, ***p < 0.001. Model 1: dependent variable: depression; independent variables: demographic control variables. Model 2: dependent variable: depression; independent variables: demographic control variables and retirement. Model 3: dependent variable: social participation; independent variables: demographic control variables and retirement. Model 4: dependent variable: depression; independent variables: demographic control variables, retirement and social participation.

**Figure 1:**
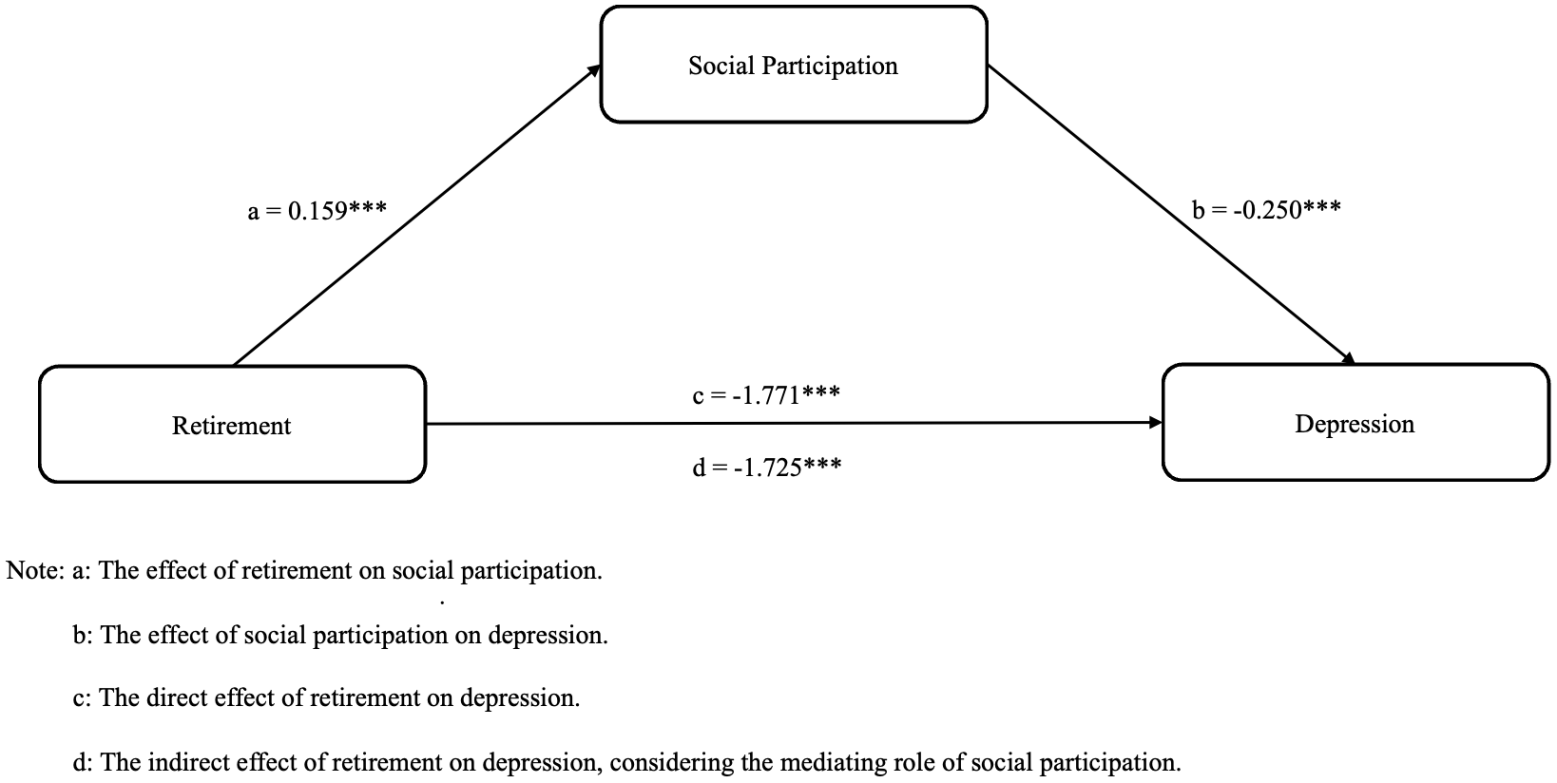
The framework for social participation' mediating role in the relationship between retirement and depression. ***P<0.001.

**Table 4** demonstrated that bootstrap methods were utilized once more to assess the mediating influence of social participation skills (56). In the mediation model where, social participation serves as a mediator, the direct impact of retirement on depression was -1.725 (95% CI = [-2.108, -1.337]), the indirect effect via social participation was -0.046 (95% CI = [-0.075, -0.017]), and the total effect was -1.771 (95% CI = [-2.153, -1.384]). The findings revealed that social participation partly mediated the relationship between retirement and depression, with the mediating impact constituting 2.6% of the overall effect. We construct a mediating effect framework to clarify the outcomes of hypothesis testing on social involvement, retirement, and depression (**Figure 1**).

**Table 4:** Bootstrap analysis of the mediating effects of social participation.

Path	β	BootSE	95% CI		Percentage of total effect (%)
			Lower	Upper	
Total effect	-1.771	0.196	-2.153	-1.384	100
Direct effect	-1.725	0.197	-2.108	-1.337	97.40
Indirect effect	-0.046	0.015	-0.075	-0.017	2.60

CI, confidence interval.

## 4 Discussion

This study utilized 2020 CHARLS data to analyze direct and indirect connections among variables by examining an integrated model of the influences of retirement and social participation on older adults’ depression, aiming at clarifying potential mechanisms that improve the comprehension of the health status of the elderly in China. The findings indicate that retiring markedly decreases depression and improves the mental health of older adults. Research findings in Asia indicate that retiring can significantly impact the health state of people over 60 years (49, 56). Nonetheless, our study found results that contradict those of previous research. The findings in this research confirm hypothesis H1, indicating that retirement decreases the likelihood of depression in older individuals (50, 57). This is because retirement frees individuals from the stresses of work, and due to reduced stress and demands, older people have more time for rest, personal interests, and family life, which improves depression (18).

Retirement directly affects depression and is amplified by the mediating factor of social participation. According to our research, older adults’ depression is directly impacted by their social participation. In agreement with hypothesis H2, social participation mediates the connection between retirement and depression in this demographic, suggesting that retirement may indirectly impact depressive symptoms through social participation. This supports existing literature and activity theory, indicating that neighborhood participation may reduce depression throughout the transition to retirement (18, 58). After retirement, participating actively with relatives and close friends may significantly reduce the probability of depressed symptoms (59).

Meanwhile, interpersonal relationships are intimately correlated with depression, and good interpersonal relationships can significantly improve subjects’ depression (60). The ability of older individuals to adapt and redefine their roles after retirement promptly and adapt to retirement life is the key to determining the post-retirement quality of life for senior individuals and preventing the emergence of psychological problems such as retirement syndrome (e.g., anxiety, depression, and loneliness). Although there is an increase in social participation after retirement from a group perspective, the reason for this may be that after retirement, there is more time and energy to participate in community activities, volunteerism, or other social activities that contribute to increased social interaction and support. However, this trend does not apply to all individuals. Some individuals who become more isolated after retirement may face higher depression risks. For this group of people, by increasing social participation after retirement, individuals can gain more social support and emotional connection, which is essential for improving depression (61). The strength of this paper is that, first, this study introduces an analytical structure to clarify the connection between retirement, social activity, and older adults’ depression. Our study framework supports the validity of activity theory and refutes the social disengagement hypothesis by demonstrating the impact of retirement on the depression of older adults. Also, this study enhances activity theory by investigating a novel mechanism linking retirement to the depression of older individuals, namely the mediating influence of social participation. Second, this study is focused on China, where the mandatory retirement age policy minimizes changes in retirement timing. The relatively fixed retirement age contrasts with countries where the retirement age is more flexible and can be influenced by factors such as personal health, financial readiness, or job satisfaction. By studying a population with a standardized retirement age, our study minimizes the confounding effects of self-selected retirement timing and can more clearly examine the causal relationships between retirement, social participation, and depressive symptoms. Finally, this research serves as a foundation for formulating social policy. Social participation may substantially enhance both the mental and physical condition of the elderly, including the improvement of physical functioning (62) to alleviate symptoms of depression (33). Social policy should thus make it easier for senior citizens to participate in social activities. Therefore, promoting the social participation of older adults in social activities, thereby promoting social interactions among older individuals (63), strengthening the social participation of older individuals, and encouraging their mental well-being.

However, this study has several shortcomings and requires additional development. Firstly, the results of this study were not entirely immune to the impact of external factors, like mood and life stress, as all variables were self-reported by the participants. Secondly, in addition to retirement and social participation, different levels of education may also affect health perceptions, ability to access social participation and thus depression in older adults (64), so future research could further explore the impact of additional factors on depression. Thirdly, this study was performed on older Chinese adults. Based on differences in national, regional, and cultural policies, the findings presented in this work are particular to the Chinese context. The influence of retirement on mental health may be contextually influenced, so caution is needed when applying our findings to other nations. Fourthly, due to the differences between rural and urban retirement systems, the findings may apply mainly to the urban worker group. Also, the household type may affect the frequency of social interaction, life stress, etc., and thus depression (65). Therefore, future research should further explore the differences between rural and urban groups to fully understand the impact of retirement policies or household type on depression among older adults from different backgrounds. Fifthly, depression was measured using the CES-D scale, and there are certain limitations to using a self-reported measure such as the CES-D instead of a clinical assessment. At the same time, this study did not make a clinical diagnosis of major depressive disorder (MDD). Future studies could shed further light on the impact of retirement on symptoms of different degrees of depression. Meanwhile, the research was constrained by its cross-sectional methodology, so future studies will use a longitudinal method to more accurately delineate the association. Finally, although China’s retirement policy is mandatory, there are cases where people do not apply for a pension, delay retirement, or continue working. For example, in rural areas, some farmers may continue to work in agriculture due to financial needs, while some professionals, technicians, or managers in urban areas may continue to work through re-employment or flexible employment. Although we aimed to control for key confounders, some potential factors, such as pre-retirement occupational experience or lifetime earnings, could not be fully accounted for due to data availability. Future research should consider more detailed measures of occupational history and post-retirement financial security to more fully understand this relationship. Without limits, this analysis possesses significant implications.

## 5 Conclusion

This study demonstrates that retirement significantly decreases depression. Non-retired older individuals exhibit a higher propensity for depression compared to their retired partners. We also confirmed that retirement affects depression in older adults directly and indirectly through social participation.

In addition, China’s mandatory retirement age is the lowest globally. Although increasing this age is viewed as a significant policy remedy for the continued viability of pensions and other economic issues related to aging, it causes more significant consideration regarding the potential subjective welfare detriment to older adults. Older adults constitute a significant demographic in human society, and their condition and mental health are intricately connected to societal stability and progress (66). Based on the findings of this paper, there is also a need for supportive policies related to flexible retirement policies, higher pension replacement rates, and other relevant policies to minimize the negative impacts on older adults.

Also, against the backdrop of an aging population and social change, it is essential to highlight mental health care delivery for older adults by improving the state of their minds, mainly through the beneficial impact of participating in social activities. The government and society should target interventions, provide treatment and mental support to those at elevated risk of depression, and cultivate community interaction to enhance their well-being and reduce their depression. It also provides a scientific basis for formulating effective public health intervention strategies. In addition, this research can also provide a reference for other developing countries to deal with an aging population.

## Data Availability

The datasets presented in this study can be found in online repositories. The names of the repository/repositories and accession number(s) can be found below: https://charls.pku.edu.cn/en/.
